# A Review of the Genus *Serratella* Edmunds, 1959 in China with Description of a New Species (Ephemeroptera: Ephemerellidae) †

**DOI:** 10.3390/insects13111019

**Published:** 2022-11-04

**Authors:** Manqing Ding, Luke M. Jacobus, Changfa Zhou

**Affiliations:** 1 The Key Laboratory of Jiangsu Biodiversity and Biotechnology, College of Life Sciences, Nanjing Normal University, Nanjing 210023, China; 2 Division of Science, Indiana University Purdue University Columbus, 4601 Central Avenue, Columbus, IN 47203, USA

**Keywords:** morphology, generic definition, comparison, taxonomy, *Serratella*, *Serratella acutiformis* sp. nov., mayfly

## Abstract

**Simple Summary:**

Mayflies in the Holarctic genus *Serratella* Edmunds (Ephemeroptera: Ephemerellidae) are common aquatic insects in some places. Their nymphs can live in a variety of aquatic habitats, ranging from wide rivers to narrow creeks, and their imagos sometimes occur in mass emergences. Thus, *Serratella* species are common in aquatic insect collections. However, both their immature and imaginal stages are difficult to identify based on morphology because of confused generic definitions and poor species descriptions. This situation is especially bad in China. Historically, fifteen species have been reported from this country, but more than half of them (eight) have been moved to other genera. The remaining seven, including species both endemic to China and species also occurring elsewhere in the Palearctic region, have never been compared and studied systematically. The present paper provides detailed figures and diagnostic characters of five species (one of the other two is excluded from this genus; one is synonymized) and a new species from Western China. This is the first comprehensive and detailed study of *Serratella* diversity in China, which provides valuable information and character candidates to identify the genus and helps understand its phylogenetic position in the family.

**Abstract:**

Species in the genus *Serratella* Edmunds, 1959 from China have never been compared and photographed systematically. Six valid Chinese *Serratella* species are recognized and revised in this paper. Among them, the imagos of *S. brevicauda* Jacobus et al., 2009 are unknown; the nymph of this species has a stout, strong body, with remarkably short caudal filaments and maxillary palpi. In contrast, only the imago stage of *Serratella fusongensis* (Su and You, 1988) (=*Serratella longipennis* Zhou et al., 1997, **syn. nov.**) is known; it has relatively long penes with small dorsal projections. The nymphs of *S. setigera* Bajkova, 1967 have small abdominal tergal spines but distinct, stout, blunt bristles on the spines, and the apexes of the male penes are round and shallowly divided. The fourth species, *S. acutiformis* **sp. nov.**, which was collected from Western China, has sharp penial apexes (imagos) and large abdominal spines (nymphs). Unlike the former four species, *S. ignita* (Poda, 1761) and *S. zapekinae* Bajkova, 1967 has sub-quadrate penes without prominent dorsal projections. The nymph of *S. ignita* has lateral hair-like setae on the caudal filaments, while the nymph of *S. zapekinae* lacks such setae but has pairs of tubercles on the head and pronotum. Some characters used in the generic delineation of the genera *Ephemerella* Walsh, 1862 and *Serratella*, such as nymphal maxillary palpi and hair-like setae on caudal filaments as well as features of the imaginal penes and forelegs, are varied in Chinese species. However, all species in this paper have bifurcate ventral lamellae of gill VI. Our work highlights a need for further comparative systematic study of the genera *Serratella*, *Ephemerella*, and another related genus *Torleya* Lestage, 1917.

## 1. Introduction

The ephemerellid taxon *Serratella* was established originally as a subgenus of *Ephemerella* Walsh, 1862 by Edmunds in 1959 [1], who designated *Ephemerella serrata* Morgan, 1911 [2] as the type species to include a morphologically diverse group of North American and Eurasian species (type species of *Ephemerella*: *Ephemerella excrucians* Walsh, 1862 [3], designated by Eaton in 1868 [4]), nearly half of which have been moved to other genera since then. Jacob [5] provided a study of European species, but almost all of the species subsequently were moved to other genus groups. Despite this problematic taxonomic past, *Serratella* is now widely regarded as a genus containing just under 20 species globally, based on the generic definition by Jacobus and McCafferty [6]. However, this proposal was not followed by some researchers. For example, Bauernfeind and Soldán [7] adopted a different understanding of this taxon, leaving a common and widely distributed species, *Ephemerella ignita* (Poda, 1761) [8], outside *Serratella*; Kluge [9,10] suggested that this genus is close to *Torleya* Lestage, 1917 [11] or even just a subgenus of the latter. Edmunds [1] previously suggested that the two genera may eventually prove to be inseparable. It is believed here that with more detailed descriptions of more species, some of the dust raised around *Serratella* will be cleared.

Fifteen species have been included in the genus *Serratella* in recent history in China [6,12,13,14,15,16,17,18,19,20,21,22,23,24,25]. Later works (Table 1) on this group transferred and synonymized eight of them [6,19,26,27,28,29]. However, the exact morphologies of the remaining seven species have not been compared systematically.

Among these seven species of Chinese *Serratella*, *S. ignita* (Poda, 1761) and *S. zapekinae* Bajkova, 1967 are closely similar to each other. The figures of penes in the key of Tshernova et al. are almost the same [30], and they were distinguished by the colors of the legs in the same key. So, their actual differences need to be presented in greater detail and with pictures. At the same time, the generic placement of *S. ignita* has been unstable and inconsistent. It has relatively recently been placed in the genus *Ephemerella* by Bauernfeind and Soldán [7], having been for many years, following older work of Eaton [31], placed in *Serratella*, by Jacobus and McCafferty [6] and Jacob [5], and in *Torleya*, by Kluge [9,10]. Thus, the determination of its exact systematic position requires further detailed study.

In the past decade, thousands of *Serratella* specimens have been collected from all over China. In 2021, four males, five females, and some subimagos of a new species were reared from mature nymphs. In addition, the seldom collected species *S. fusongensis* Su and You, 1988 and *S. longipennis* Zhou et al., 1997 were also found, but only in the stages previously known. The species *S. xiasimaensis* You, 1987 was depicted with four-segmented forceps in the original figures [14], and its holotype and paratypes cannot be located currently; thus, it is not included in our present study. So, six species are compared here. We emphasize that this is a regional study for China, and it includes only species recorded from this area. Conclusions and diagnoses are applicable only to this geographic area. However, their detailed characters may provide some insights into the systematics of the genus *Serratella*.

## 2. Materials and Methods

The nymphs were collected using hand nets. Some imagos and subimagos were reared from mature nymphs in a plastic container covering a nylon net indoors. The associations between imagos and nymphs were also confirmed by their COI sequences (GenBank accession numbers: OP737501, OP737502, OP737503). 

All specimens were examined under a stereo microscope (Mingmei Photoelectric, MZ81, Guangzhou, China) and photographed with a digital camera (Single Lens Reflex, Guangzhou, China). Some small structures, such as mouthparts, claws, and gills, were placed on temporary slides with ethanol to be observed and photographed under a microscope camera (Nikon Eclipse 50i, Tokyo, Japan). Eggs were dissected from mature female nymphs and photographed with a scanning electron microscope (Apreo 2S, Thermo Fisher Scientific Company, Waltham, MA, USA). All structures except eggs and gills were preserved in alcohol after the photographs were taken.

The materials mentioned in this study were deposited in the Mayfly Collection, College of Life Sciences, Nanjing Normal University (NNU), China.

The specimens were stored in ethanol (around 85%).

We used the morphological species concept.

## 3. Results

### 3.1. Serratella Acutiformis Zhou **sp. nov.**

Distribution: China (Sichuan, Yunnan).

**Figure 1:**
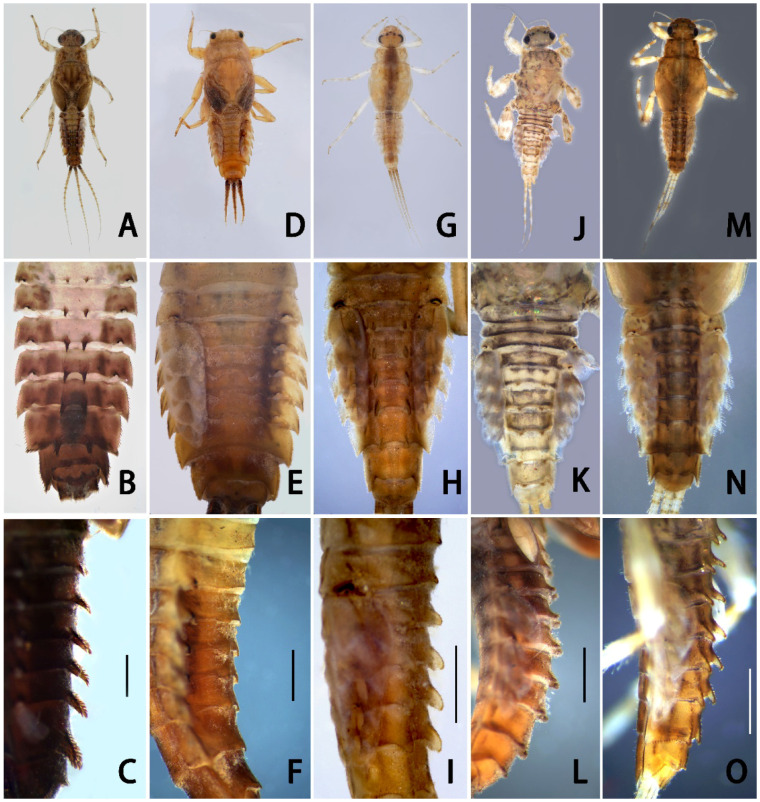
Nymphal habitus and abdomens of *Serratella* spp. of China: (**A**–**C**) *S. acutiformis* **sp. nov.**; (**D**–**F**) *S. brevicauda*; (**G**–**I**) *S. ignita*; (**J**–**L**) *S. setigera*; (**M**–**O**) *S. zapekinae*. Scale bars: 0.5 mm.

**Figure 2:**
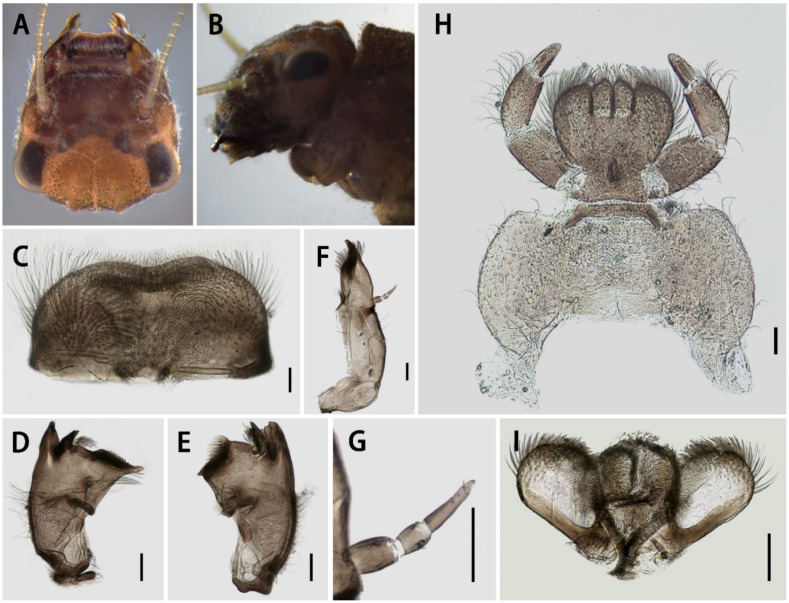
Head and mouthparts of *Serratella acutiformis* **sp. nov.**: (**A**) head (dorsal view); (**B**) head (lateral view); (**C**) labrum; (**D**) left mandible; (**E**) right mandible; (**F**) maxilla; (**G**) maxillae palp; (**H**) labium; (**I**) hypopharynx. Scale bars: 0.1 mm.

**Figure 3:**
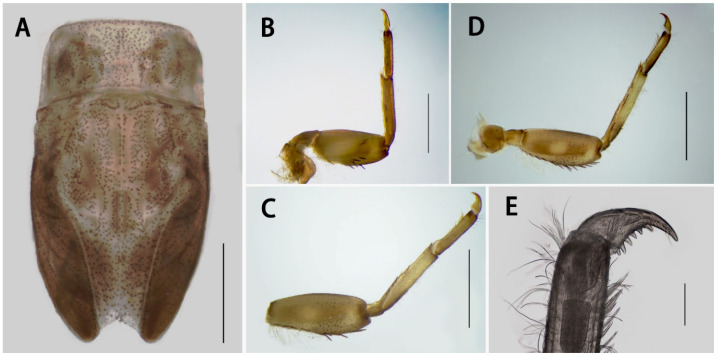
Thoracic structures of *S. acutiformis* **sp. nov.**: (**A**) thoracic nota; (**B**) foreleg; (**C**) midleg; (**D**) hindleg; (**E**) claw of foreleg. Scale bars: A, B, C, D = 1 mm; E = 0.1 mm.

**Figure 4:**
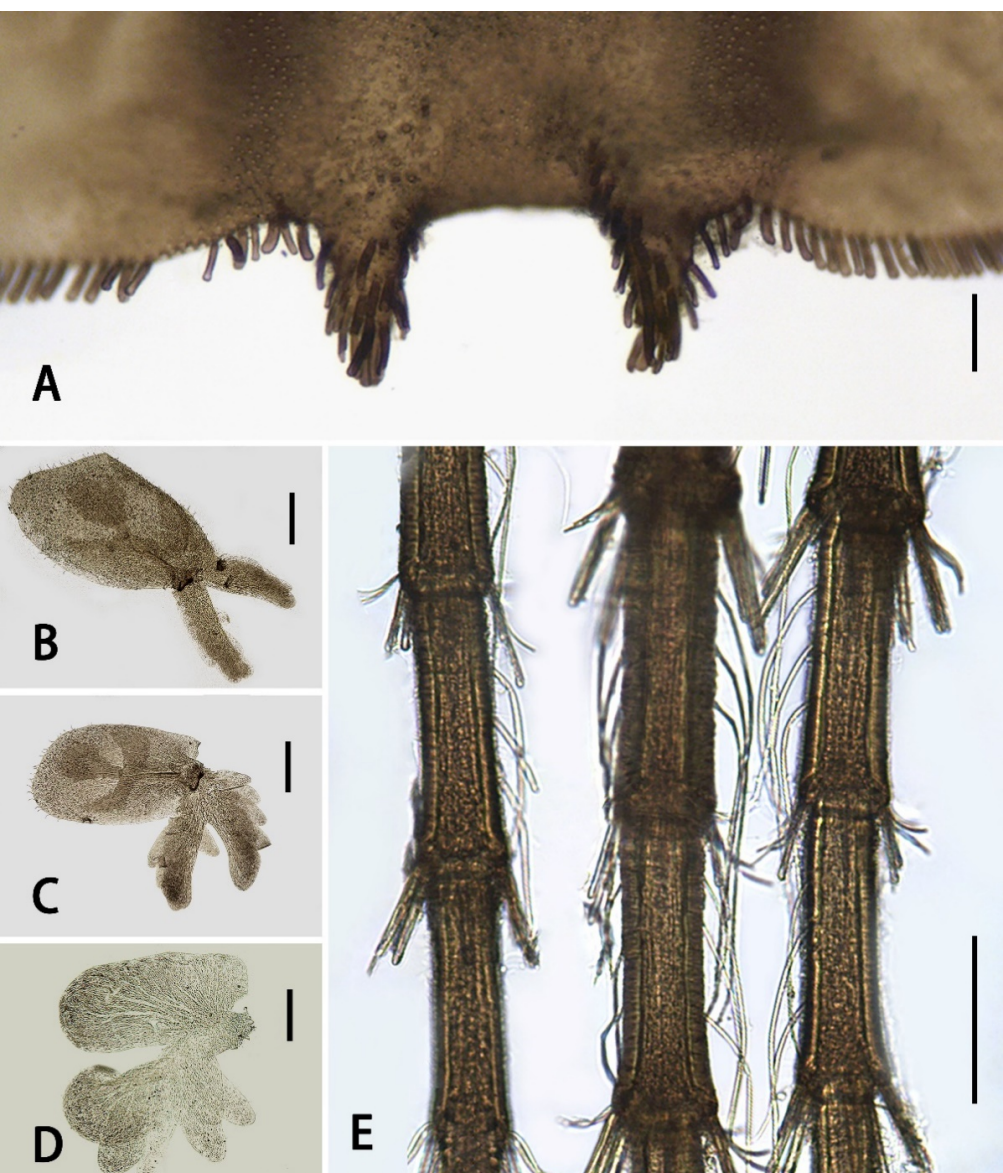
Abdominal structures of *S. acutiformis* **sp. nov.**: (**A**) spines on terga VIII; (**B**) gill III; (**C**) gill VI; (**D**) gill VII; (**E**) median part of caudal filaments. Scale bars: A, D, E = 0.1 mm; B, C = 0.2 mm.

Description of nymph (Figure 1A–C and Figure 2, Figure 3 and Figure 4): Body length 6.0–7.0 mm, caudal filament length 5.0–6.0 mm, terminal filament slightly longer than cerci; body coarse, pale, gray to brown, washed with various tan to dark dots and markings (Figure 1A); whole dorsal surface covered with very tiny brown to dark particle-like protuberances rarely present on abdomen (Figure 1B). 

Antennae pale, with tiny hair-like setae on scapes, pedicels, and articulations of flagella. Dorsal surfaces of frons and genae with transverse row of brown to dark dots, shapes of them irregular; genae slightly expanding forwards, making anterior margin of head semi-round, with dense hair-like setae. Vertex rough, with three pairs of blunt protuberances of different sizes (Figure 2B).

Labrum slightly asymmetrical (five individuals checked for this feature), right half slightly longer than left one (Figure 2C), width ca. 2.0× length, leading margin with shallow anteromedian emargination; free margins and dorsal surface with hair-like setae, ventral surface with two tufts of sub-median setae. Mandibles with long hair-like setae on outer margin and surface, outer incisor with three blunt denticles, inner incisor with two small and acute denticles (Figure 2D,E); prostheca represented by tuft of plumose setae and strong spine; mesal margin near mola of left mandible with row of long hair-like setae. Maxillae with three apical blunt canines and two distinct dentisetae, series of bristles near dentisetae (Figure 2F); tuft of bristles on apex; outer and inner margins of maxillae with hair-like setae; three-segmented maxillary palpi about half of upper half maxillae in length, with very sparse hair-like setae (Figure 2G); length ratio of three segments of maxillary palp from base to apex = 1.0:0.5:1.25, segment I broader than segments II and III; cardo also with setae. Labial palp moderately developed with three segments (Figure 2H), both segments I and II with setae on outer margin, segment III with distinct shorter and sparser setae, length ca. 3.0× width; length ratio of them from base to apex = 1.0:0.8:0.5; both dorsal and ventral surfaces of glossae and paraglossae with dense hair-like setae; glossae long and oval, with distinct convex apex, length ca. 2.0× width; width of paraglossae 2.0× that of glossae. Lingua with subparallel lateral margins (Figure 2I), surface with tiny hair-like setae; apical margins of superlinguae with long hair-like setae.

Thorax brown, with brown to dark maculae (Figure 3A); pronotum with straight anterior, lateral and posterior margins; surface of pronotum coarse, one pair of protuberances apparent. Anterolateral angles of mesonotum at same level of lateral margin of pronotum, with very shallow crease nearby. Coxa of fore- and midlegs with larger, blunter dorsal plates than hindlegs (Figure 3B,D). Femora of all legs with three dorsal brown bands at base, middle, and sub-apex respectively, basal one-third of tibiae brown, basal and apical brown bands on tarsi (Figure 1A). Forefemora with three spines and a row of hair-like setae on outer margin (Figure 3B), subapical band of spine-like setae present, inner margin with very tiny hair-like setae; fore tibiae with hair-like setae on inner and outer margins, additional row of spine-like setae on dorsal margin, tuft of bristles on inner apex; whole surfaces of tarsi with hair-like setae, some additional spine-like setae on inner margin; length ratio of femora:tibiae:tarsi = 1.0:0.9:0.6. Middle legs and hind legs similar to forelegs in color and structure pattern but without subapical band of spine-like setae (Figure 3C,D), margins with more and longer spine-like and hair-like setae. Length ratio of femora:tibiae:tarsi of midleg = 1:0:0.8:0.6, ratio of hindleg = 1.0:1.0:0.6. Mid- and hindlegs with patellar–tibial fusion sutures. Tarsal claws of all legs similar, apex hooked (Figure 3E), with three subapical setae and seven basal denticles of different sizes.

Abdomen dark-brown, terga I–V pale (Figure 1A,B). All terga with stout spine-like setae on lateral margins (Figure 1B); terga I–II with hair-like setae on posterior margins, tergum II with additional spine-like setae on posterior margin; terga III–VIII each with a pair of acute median spines extending backwards and upwards into distinct spur- or horn-like structures (Figure 1B,C), larger progressively from anterior to posterior; spines on tergum VIII distinctly larger and more widely spaced than anterior pairs (Figure 1B and Figure 4A). All spines of abdomen with additional stout, blunt, bristle-like setae (Figure 4A), those setae also on posterior margins of terga VIII and IX, forming comb-like structures along with spines and their associated setae.

Gills III–VI similar, with dorsal lamella sub-quadrate and with trilobed markings (Figure 4B,C); ventral lamella divided into two clusters of lobes. Gills VII membranous, much smaller than others, ventral lamella comprised of several lobes (Figure 4D).

Caudal filaments with dark-brown base, fading to apex (Figure 1A). Articulations of basal half alternately with distinct spine-like bristles and hair-like setae but articulations of apical half with both kinds of setae (Figure 4E); apical half of terminal filament with additional hair-like setae on both sides, while apical half of cerci with mesal fringes only.

**Figure 5:**
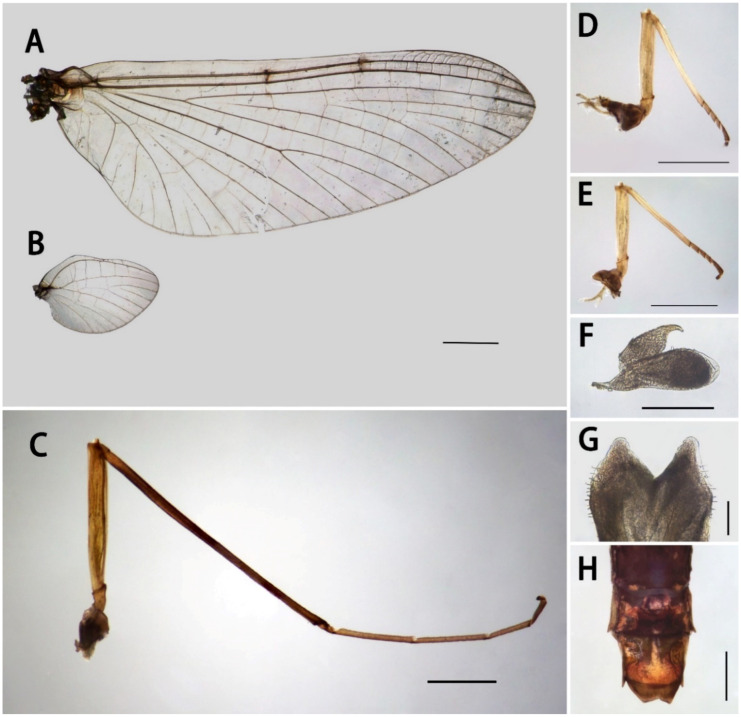
Imaginal structures of *S. acutiformis* **sp. nov.**: (**A**) forewing; (**B**) hindwing; (**C**) foreleg; (**D**) midleg; (**E**) hindleg; (**F**) claw of foreleg; (**G**) apex of penis; (**H**) terminal abdomen of female imago. Scale bars: A, B, C, D, E = 1 mm; F = 0.1 mm; G = 0.05 mm; H = 0.5 mm.

**Figure 6:**
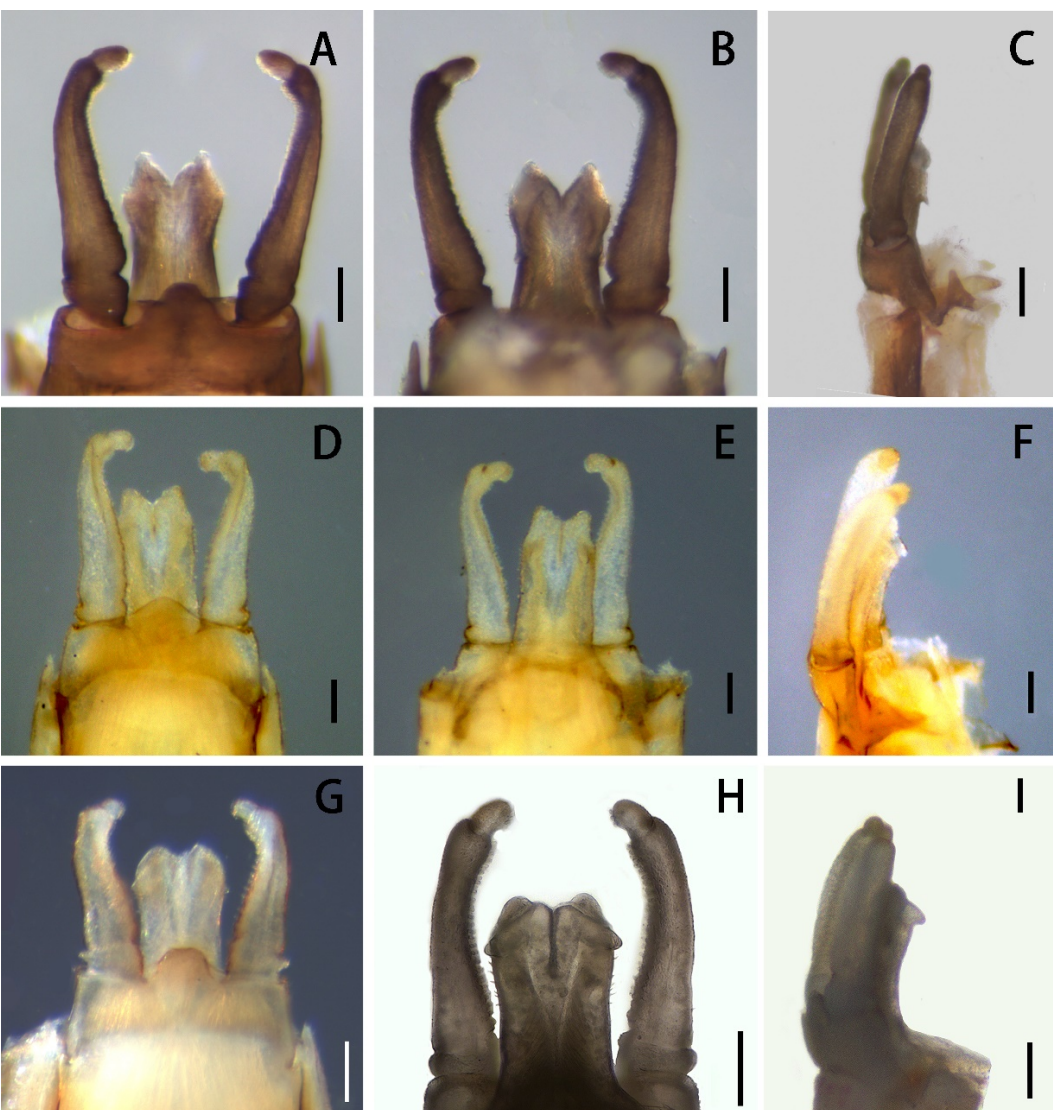
Male genitalia of *S. acutiformis* **sp. nov.** in (**A**) ventral view; (**B**) dorsal view; (**C**) lateral view. Male genitalia of *S. fusongensis* in (**D**) ventral view, (**E**) dorsal view, (**F**) lateral view. Male genitalia of *S. setigera*: (**G**) ventral view, (**H**) dorsal view, (**I**) lateral view. Scale bars: 0.1 mm.

Description of male imago (Figure 5A–G and Figure 6A–C): Body 8.0 mm, caudal filaments ca. 9.0 mm. Body brown, with pale sutures, and one pair of median pale dots on mesothorax. Compound eyes contiguous with reddish upper portion but dark lower portion; ocelli with dark bases.

Forewing 9.0–10.0 mm, transparent (Figure 5A); C and Sc areas semi-transparent; longitudinal veins pale-brown to yellowish-brown; crossveins in stigma region divided into upper and lower portion by transverse vein; MA forked at distal 1/3 point; bases of CuA and CuP close to one another, with three relatively long intercalary veins between them; fewer crossveins near margin than elsewhere; single marginal intercalary veins clear; Sc, R1, and R5 with clear bullae. Hindwing 2.0 mm, hyaline (Figure 5B); costal margin with shallow round process and concave crease. Forelegs 6.8 mm, tibiae browner than femora and tarsi (Figure 5C); length ratio of forefemora:tibiae:tarsi = 1.0:2.0:1.6; tarsal segments arranged in decreasing order as 2, 3, 4, 5, 1, basal one very short. Mid- and hindlegs similar (Figure 5D,E); tarsi slightly browner than femora and tibiae, latter two pale. Length ratio of midfemora:tibiae:tarsi = 1.0:1.2:0.5; tarsal segments arranged in decreasing order as 4, 1, 2, 3; length ratio of hindfemora:tibiae:tarsi = 1.0:1.3:0.5; tarsal segments arranged in decreasing order as 4, 1, 2, 3. Claws of all legs similar, one blunt, one acute (Figure 5F).

Abdomen uniformly reddish-brown to brown; terga VIII and IX with short but clear posterolateral projections. Styliger plate with distinct round median projection (Figure 6A). Segment II of forceps slightly bent inwards, with shallow subapical crease (Figure 6A,B); forceps segment III length less than 2.0× width, only 1/6–1/7× second one in length; all segments of forceps with tiny projections along inner margins. Penes ca. 0.6× length of forceps, with pointed apexes and median V-shaped cleft between them; each penis with some setae on lateral surface (Figure 5G) and dorsal subapical projection, visible in ventral and dorsal views (Figure 6A–C). Caudal filaments brown, articulations slightly darker, with tiny setae.

Description of female imago (Figure 5H): Body 7.0–7.5 mm, caudal filaments 9.0 mm. Forewings 10.0–10.5 mm. Forelegs 4.3 mm; length ratio of forefemora:tibiae:tarsi = 1.0:1.5:0.8, tarsal segments arranged in decreasing order as 2, 5, 3, 4,1; ratio of midleg = 1.0:1.3:0.6, tarsal segments arranged in decreasing order as 4, 1, 2, 3; length ratio of hindfemora:tibiae:tarsi = 1.0:1.0:0.4, tarsal segments arranged in decreasing order as 1, 4, 3, 2. Subgenital plate slightly extended, subanal plate with shallow V-shaped posterior margin (Figure 5H). Terga VIII–IX with clear postolateral projections.

Description of male subimago: Wings semi-transparent. Forelegs 3.4 mm; length ratio of forefemora:tibiae:tarsi = 1.0:1.4:1.0; tarsal segments arranged in decreasing order as 2, 3, 5, 4, 1. Length ratio of midfemora:tibiae:tarsi = 1.0:1.2:0.5, tarsal segments arranged in decreasing order as 1, 4, 2, 3. Length ratio of hindfemora:tibiae:tarsi = 1.0:1.0:0.6; tarsal segments arranged in decreasing order as 4, 1, 2, 3.

Description of female subimago: Wings semi-transparent. Forelegs 3.4 mm; length ratio of forefemora:tibiae:tarsi = 1.0:1.1:0.8, tarsal segments arranged in decreasing order as 2, 3, 5, 4, 1. Length ratio of midfemora:tibiae:tarsi = 1.0:1.3:0.6, tarsal segments arranged in decreasing order as 4, 1, 2, 3. Length ratio of hindfemora:tibiae:tarsi =1.0:1.2:0.5, tarsal segments arranged in decreasing order as 4, 1, 2, 3.

**Figure 7:**
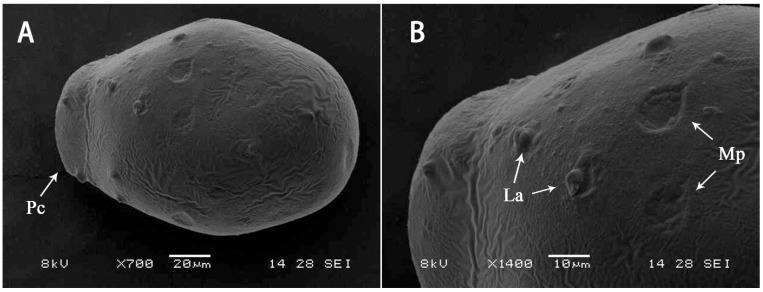
Egg of *S. acutiformis* **sp. nov.**: (**A**) overall view of egg; (**B**) detail view of egg. Abbreviations: Pc, polar cap; Mp, micropyle; La, lateral attachment structure.

Description of egg (Figure 7): Length 150–160 μm, width 100–120 μm. Drum-like in shape, with one polar cap; surface with scattered attachment structures; micropyles present.

Etymology: The specific name *acutiformis* is a Latin word meaning acute or sharp, indicating the acute apex of the imaginal penes (Figure 5G and Figure 6A) and nymphal abdominal spines (Figure 1C).

**Figure 8:**
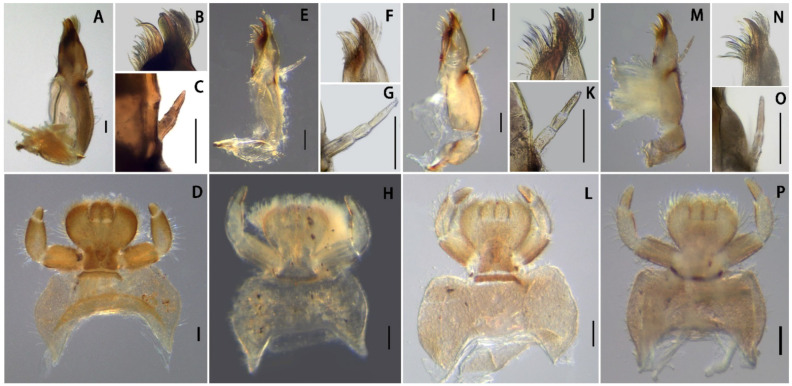
Maxillae and labia of *Serratella* spp. of China: (**A**–**D**) *S. brevicauda*; (**E**–**H**) *S. ignita*; (**I**–**L**) *S. setigera*; (**M**–**P**) *S. zapekinae*. Scale bars: 0.1 mm.

Diagnosis: The nymphs of the new species *Serratella acutiformis* **sp. nov.** resemble four other Chinese *Serratella* species in having spine-like setae on their abdominal terga and their spines (Figure 1), reduced maxillary palpi and similar maxillae (Figure 2F,G and Figure 8), subapical bands of spine-like setae on the forefemora (Figure 3B), claws with basal denticles and apical setae (Figure 3E), and divided ventral lamellae of gills VI (Figure 4C). In males, this new species is similar to *S. fusongensis* and *S. setigera* in having a relatively straight segment II of the genital forceps, nearly round segment III; dorsal projections on penes (Figure 6); and subequal foretibiae and tarsi (Figure 5C). It is a definite *Serratella* species according to the definition of this genus provided by Jacobus and McCafferty [6] and Bauernfeind and Soldán [7].

Our new species can be distinguished from other Chinese species by the following characters. In the nymph, it has distinct abdominal spines on terga III–VIII (Figure 1B,C) and moderately developed maxillary palpi (Figure 2F,G) (longer than those of *S. brevicauda* but shorter than those of other species (Figure 8)). Its paired tergal spines have denser distributions of bristle-like setae (Figure 4A). Furthermore, the apical halves of its caudal filaments have lateral hair-like setae (Figure 4E), and its head with three pairs of protuberances is also useful with respect to identification (Figure 2A,B). The male imago has longer foretibiae than tarsi (Figure 5C), acute penial apexes, and ventrally visible subapical projections on the dorsal sides of the penes (Figure 6A–C).

**Figure 9:**
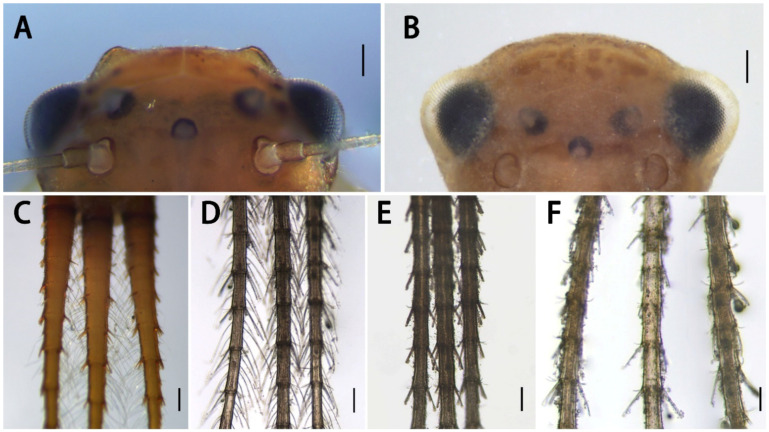
Nymphal structures of *Serratella* spp. of China: (**A**) vertex of *S. zapekinae*; (**B**) vertex of *S. ignita*. Caudal filaments of: (**C**) *S. brevicauda*; (**D**) *S. ignita*; (**E**) *S. setigera*; (**F**) *S. zapekinae*. Scale bars: 0.1 mm.

The nymph of *Serratella setigera* is somewhat similar to this new species in having abdominal spines on terga III–VIII, but the spines of the former are much shorter (Figure 1K,L). Dissimilarly, *S. setigera* has no protuberances on the head and pronotum, nor lateral setae along caudal filaments (Figure 9E), but has more setae covering its nymphal body (Figure 1J). Moreover, the two species have different imaginal penes (Figure 6A–C,G–I).

The nymphs of this new species resemble *S. brevicauda* in having strong bodies (Figure 1A,D) and lateral setae on caudal filaments (Figure 4E and Figure 9C). However, the latter species has obviously shortened tails (Figure 1D), distinctive maxillary palpi (Figure 8C), and apical segments of labial palpi (Figure 8D). The terga have shorter and blunter spines (Figure 1E,F). The imagos of the latter are still unknown.

On the other hand, the males of the new species are similar to *S. fusongensis* in penial shape (Figure 6A–F). However, the penes of the latter species are more narrowly divided, and the dorsal subapical projections are smaller, which cannot be seen in the ventral view (Figure 6D–F). The nymphs of *S. fusongensis* are still unknown.

**Figure 10:**
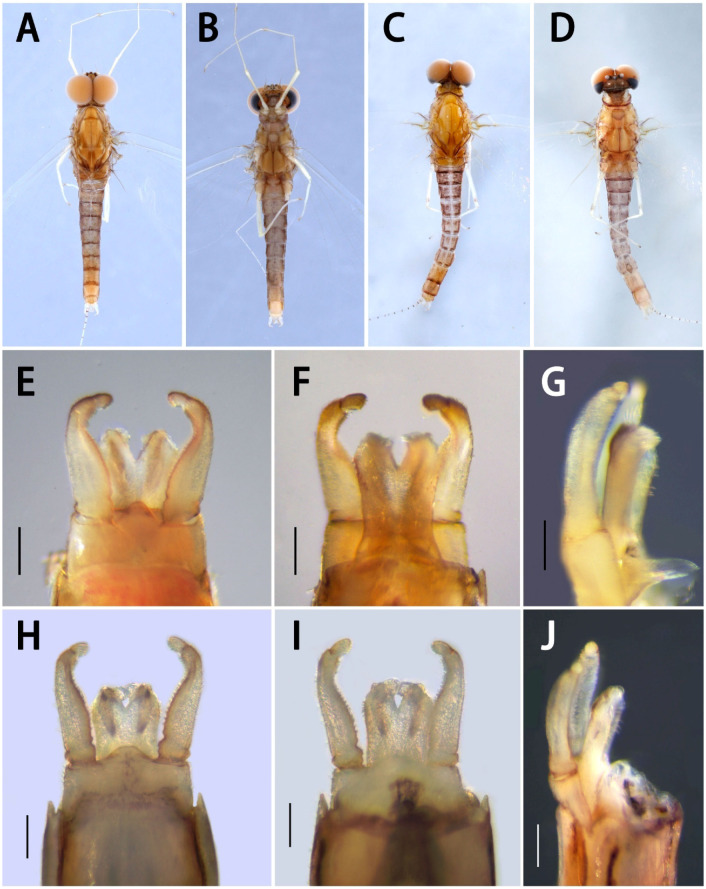
Imagos of *S. ignita*: (**A**) habitus (dorsal view); (**B**) habitus (ventral view); (**E**) genitalia (ventral view); (**F**) genitalia (dorsal view); (**G**) genitalia (lateral view). Imagos of *S. zapekinae*: (**C**) habitus (dorsal view); (**D**) habitus (ventral view); (**H**) genitalia (ventral view); (**I**) genitalia (dorsal view); (**J**) genitalia (lateral view). Scale bars: 0.1 mm.

In contrast to the penes of *S. acutiformis* **sp. nov.**, which has divided apexes and distinct dorsal projections (Figure 5G and Figure 6A–C), the penes of *S. ignita* (Figure 10E–G) and *S. zapekinae* (Figure 10H–J) have no prominent dorsal projections and are sub-quadrate in shape. Although the nymphs are similar with respect to the labial palpi (Figure 2H and Figure 8H,P), the new species has a shorter apical segment of the maxillary palp (Figure 2G and Figure 8G,O), and larger abdominal spines than the latter two species (Figure 1C,I,O). In addition, the outer sides of the cerci of the new species *S. acutiformis* **sp. nov.** have no hair-like setae (Figure 4E), though these setae are present on the cerci of the species *S. ignita* (Figure 9D). The caudal filaments of *S. zapekinae* have no lateral hair-like setae (Figure 9F). Furthermore, nymphs of both *S. acutiformis* **sp. nov.** and *S. zapekinae* have protuberances on their heads and pronota, but the former species has three pairs of protuberances on the head (Figure 2B), while the latter has one pair only (Figure 9A).

According to Edmunds [1] and Allen and Edmunds [32], the North American *Serratella* species have no lateral hair-like setae on their caudal filaments and their penial apexes are very blunt. The European *Serratella* species in the book of Bauernfeind and Soldán [4] is similar to the American ones.

Materials examined: Holotype: Male imago, Mugetso Scenic Area, Kangding City, Ganzi Tibetan Autonomous Prefecture, Sichuan Province (101.543859° E, 30.113633° N), 24-VII-2021, collected by Xu-Hong-Yi Zheng, Peng-Xu Mu. Paratypes: One male imago, five female imagos, one male subimago, and eight female subimagos, same as the holotype. Other materials: 1 nymph ecdysis, same as the holotype; 1 male imago, Yingjing County, Yaan city, Sichuan Province (102.5396° E, 29.375611° N), 21-VII-2021, collected by Xu-Hong-Yi Zheng, Peng-Xu Mu; 118 nymphs, Niujie Township, Eryuan County, Dali Prefecture, Yunnan Province (100.01290° E, 26.20905° N), 8-VII-2008, collected by Hui Xie, Yan-Yan Jia, Ping Chen; 55 nymphs, Zhaojue County, Liangshan Yi Autonomous Prefecture, Sichuan Province (102.55734° E, 27.88159° N), 4-VII-2005, collected by Chang-Fa Zhou.

### 3.2. Serratella brevicauda Jacobus, Zhou and McCafferty, 2009

*Serratella brevicauda* Jacobus, Zhou and McCafferty, 2009: 53 [24], figures 1–3 (nymph). Holotype: Nymph, from Yunnan, China.

*Serratella brevicauda*: Zhou, 2013: 181 [29]; Zhou et al., 2015: 229 [25].

Distribution: China (Yunnan, Shaanxi).

Descriptions: See Jacobus, Zhou, and McCafferty, 2009 [24].

Diagnosis: This species was described using nymphs only. Its nymphs have remarkedly reduced caudal filaments (Figure 1D), maxillary palpi (Figure 8A–C), and apical segment of the labial palpi (Figure 8D). In addition, the bodies of this species are very stout and strong, and their caudal filaments have lateral setae on the apical half (Figure 9C). However, its maxillae are similar to other species in the present paper, and its nearly oval head shape is also similar to that of the new species *S. acutiformis* **sp. nov.**

Remarks: Although the imagos of this species are still unknown, it may be a species of another genus given the combination of its stout body (Figure 1D), shortened but strong tails with long hair-like setae and short spine-like and bristle-like setae (Figure 9C) and distinctive maxillary palpi (Figure 8C), and indistinctive spines on abdominal terga (Figure 1E,F).

Materials examined: Paratypes: 40 nymphs, Niujie Township, Eryuan County, Dali Prefecture, Yunnan Province, 24-V-1996, collected by Chang-Fa Zhou; 1 nymph, Heihe Forest Park, Zhouzhi County, Shaanxi Province (108.032311° E, 33.913533° N), 12-IV-2015, collected by Kai-Li Liu, Jing-Xia Zhao; 1 nymph, Huyi County, Xi’an City, Shaanxi Province (108.46606° E, 33.8555° N), V-2012, collected by Sheng Xu, Zhao Xie.

### 3.3. Serratella fusongensis (Su and You, 1988)

*Ephemerella (Ephemerella) fusongensis* Su and You, 1988: 64 [15], figures 10–14 (male). Holotype and paratypes: Male, from Fusong County, Jilin (transferred to *Serratella* by Jacobus and McCafferty, 2008: 241 [6]).

*Ephemerella (Ephemerella) fusongensis*: You and Gui, 1995: 136, figure 146 (male) [33]. 

*Ephemerella fusongensis*: Quan et al., 2002: 248 [23]; Kluge, 2004: 315 [11] (incertae sedis).

*Serratella fusongensis*: Zhou et al., 2015: 229 [25].

*Serratella longipennis* Zhou et al., 1997: 269 [18], figures 6–9 (male, female). Holotype and paratypes: Male, female, from Bai-Yun-Shan, Henan, China; Kluge, 2004: 315 [11] (incertae sedis); Jacobus and McCafferty, 2008: 241 [6]; Bauernfeind and Soldán, 2012: 474 [7]; Zhou, 2013: 181 [29]; Zhou et al., 2015: 230 [25] **syn. nov.**

Distribution: China (Northern part).

Descriptions: See Su and You, 1988 [15], or Zhou et al., 1997 [18].

Diagnosis: The males of this species can be differentiated by the longer penes which, further, have smaller apical projections (Figure 6D–F). The tip of the penis in some individuals extended inwards to form a small dorsal projection. The two penes are almost fused in full length, with a broad V-shaped emargination between them.

Remarks: We examined the holotypes of two species, *S. fusongensis* and *S. longipennis*, and found that they are conspecific. In the original pictures, Su and You [15] showed or concentrated on the division between the two penes, while Zhou et al. [18] focused on their apical projections. These inaccurate illustrations caused some misunderstandings, and this synonymy is the result. 

Based upon its penes, this species might be a member of the genus *Torleya*, like *Torleya mikhaili* Tiunova, 1995 [34]. Its exact status will be clarified when the nymph can be described.

Materials examined: Holotype of *S. fusongensis*: Male imago, Fusong County, Baishan City, Jilin Province, 18–25-VII-1984, collected by Feng Peng, Ye Li. Paratypes: One male imago, same as holotype. Holotype and two paratypes of *S. longipennis*: Male imago and two female imagos, Baiyun Mountain, Song County, Luoyang City, Henan Province, 16-VII-1996, collected by Chang-Fa Zhou, Bei-Xin Wang. Other materials: 2 male imagos, Danbao, Wen County, Gansu Province, 30-VII-2000, collected by Chang-Fa Zhou, Qiang Xie; 1 male imago and 31 female imagos, Balan River, Langxiang Town, Tieli City, Heilongjiang Province, 5-VIII-1993, collected by You-Wen Li, Chang-Hai Sun; 14 male subimagos and 1 female subimago, Nanchang City, Jiangxi Province, 11-VI-1980; 2 female imagos, 1 male imago, with 11 female subimagos and 1 male subimago, Xunyang Ba Town, Ningshan County, Ankang City, Shaanxi Province, 2-VII-1982.

### 3.4. Serratella ignita (Poda, 1761)

*Ephemera ignita* Poda, 1761: 97 [8] (male). Holotype: Imago, from Europe. 

*Ephemera erythrophalma* Schrank, 1798: 197 (synonymized by Eaton, 1871: 98 [35]).

*Ephemera fusca* Stephens, 1835: 58 [36] (synonymized by Eaton, 1871: 98 [35]).

*Ephemerella diluta* Stephens, 1835: 58 [36]; Walker, 1853: 545 [37]; Hagen, 1863: 19 [38] (synonymized by Eaton, 1871: 98 [35]).

*Ephemera apicalis* Stephens, 1835: 59 [36]; Walker, 1853: 544 [37] (synonymized by Eaton, 1871: 98 [35]).

*Ephemera rufescens* Stephens, 1835: 59 [36] (synonymized by Eaton, 1871: 98 [35]).

*Ephemera rosea* Stephens, 1835: 59 (synonymized by Eaton, 1887: 126 [31]).

*Baetis obscura* Stephens, 1835: 65 [36] (synonymized by Eaton, 1871: 98 [35]).

*Potamanthus erythrophtalmus* Pictet, 1843: 222 [39], plates 29, 30 (nymph, imago); Walker, 1853: 544 [37]; Hagen, 1863: 21 [38] (synonymized by Eaton, 1871: 98 [35]).

*Potamanthus gibbus* Pictet, 1844: 226 [39], plates 31, 32 (imago, subimago); Walker, 1853: 545 [37] (synonymized by Eaton, 1887: 126 [31]).

*Potamanthus aeneus* Pictet, 1844: 229 [39], plate 33 (egg, nymph, subimago, imago); Walker, 1853: 545 [37] (synonymized by Eaton, 1887: 126 [31]).

*Potamanthus apicalis* Pictet, 1844: 236 [39]; Walker, 1853: 544 [37] (synonymized by Eaton, 1871: 98).

*Potamanthus dilectus* Pictet, 1844: 236 [39]; Walker,1853: 545; Hagen, 1863: 19 [38] (synonymized by Eaton, 1871: 98 [35]).

*Potamanthus roseus* Pictet, 1844: 236 [39]; Walker, 1853: 545 [37] (synonymized by Eaton, 1871: 98 [35]).

*Ephemerella aenea* Eaton, 1871: 99 [35]; Meyer-Dür, 1874: 316 [40] (synonymized by Eaton, 1887: 126 [31]).

*Ephemerella gibba* Eaton, 1871: 99 [35]; Meyer-Dür, 1874: 316 [40]; Rostock, 1878: 85 [41] (synonymized by Eaton, 1887: 126 [31]).

*Ephemerella ignita*: Eaton, 1871: 98 [35], plates ii. 5 and v. 7–7a (nymph, imago); Meyer-Dür, 1874: 316 [40]; Rostock, 1878: 85 [41]; Halford 1887: 235 [42]; Eaton, 1887: 126 [31], plate xiv. a (nymph, male); Bengtsson, 1913: 286 [43], plates i. 4–5 and ii. 9 (egg); Lestage, 1917: 359 [9], figure 33 (nymph); Lestage, 1925: 245 [44], figure 6 (gill); Brekke, 1965: 12 [45]; Bajkova, 1972: 181 [46], figures 7–16 (nymph and imago); Dahlby, 1973: 251 [47]; Kluge, 1995: 40 [48]; Bauernfeind and Soldán, 2012: 466 [7].

*Ephemerella lactata* Bengtsson, 1909: 6 [49] (synonymized by Dahlby, 1973: 251 [47]).

*Ephemerella torrentium* Bengtsson, 1917: 178 [50] (synonymized by Brekke, 1965: 12 [45]).

*Ephemerella sibirica* Tshernova, 1952: 278 [51] (synonymized by Bajkova, 1972: 181 [46]).

*Ephemerella (Ephemerella) ignita*: Jacob, 1974: 5 [52].

*Drunella karasuensis* Kustareva, 1976: 58 (synonymized by Kluge, 1995: 40 [48]).

*Ephemerella (Serratella) ignita*: Tshernova et al., 1986: 138 [30], figure 61: 8 (male); Jacob, 1986: 222 [53].

*Ephemerella (Ephemerella) antuensis* Su and You, 1989: 181 [16], figures 1–27 (nymph, imago). Holotype: Imago, from China; You and Gui, 1995: 136 [33], figure 145 (synonymized by Kluge, 2004: 312 [11]).

*Ephemerella (Torleya) ignita*: Kluge, 1997: 213 [10], figure 18: 1–3 (nymph); Kluge, 2004: 312 [11].

*Serratella ignita*: Jacob 1993: 107 [5], figures 1, 2b (nymph, imago); Jacobus and McCafferty, 2008: 241 [6]; Zhou et al., 2015: 229 [25].

*Ephemerella antuensis*: Quan et al., 2002: 247 [23].

(Most of the citations above for European research can be found in Bauernfeind and Soldán, 2012 [7]). 

Distribution: Palaearctic Region.

Descriptions: See Bajkova, 1972 [46], or Su and You, 1989 [16].

Diagnosis: The nymphs are unique among Chinese species in having relatively long apical segments of maxillary and labial palpi (Figure 8E–H) and lateral hair-like setae on both sides of the caudal filaments (Figure 9D). The male imagos are remarkably similar to the species *S. zapekinae* in having sub-quadrate penes without prominent apical projections (Figure 10E–J). Three characters can be used to separate imagos of this species from *S. zapekinae*: (1) the posterior margin of the median styliger plate, which is sharply convex (Figure 10E), while that of *S. zapekinae* is less pronounced (Figure 10H); (2) its forceps with deeper subapical creases in the second segments and which are overall slightly more bent than the forceps of *S. zapekinae* (Figure 10E,H); (3) the abdomen, which is almost uniformly brown to dark-brown (Figure 10A), while that of *S. zapekinae* usually has a pale median longitudinal line (Figure 10C). In contrast, the nymphs are very different. *Serratella ignita* has hair-like setae on caudal filaments (Figure 9D) but has no protuberances on the head (Figure 9B) or pronotum, while *S. zapekinae* has a pair of protuberances on both the head (Figure 9A) and pronotum but has no lateral hair-like setae on the segments of the caudal filaments (Figure 9F).

Remarks: Bauernfeind and Soldán [7] placed this species in the genus *Ephemerella*, but Jacob [5] and Jacobus and McCafferty [6] and others have placed it in *Serratella*. Although it has lateral hair-like setae on the caudal filaments (Figure 9D), a relatively long maxillary palp in the nymph (Figure 8G), and no prominent projections on the penes (Figure 10E–G), it is regarded as a member of the genus *Serratella* in the present study for the following three reasons: (1) its gills VI are bifurcate, unlike the condition of *Ephemerella* (ventral lamella of gill VI undivided); (2) the distribution of setae on the tails and the lengths of maxillary palpi are somewhat variable among the Chinese species; and (3) the species *S. zapekinae* is very similar to this one, especially in terms of their almost identical penes.

Further, Bauernfeind and Soldán [7] reported that this species’ nymphs have lateral setae on the apical one-third of the caudal filaments. However, the Chinese nymphs have at least sparse setae along the whole length of the tails.

Materials examined: Holotype of *Ephemerella antuensis*: Male imago, Mingyue Town, Antu County, Jilin Province, 18–25-VII-1984, collected by Feng Peng, Ye Li. Paratypes: 10 female imagos, same as the holotype. Other materials: 6 male imagos, 1 female imago, Lushuihe Town, Fusong County, Jilin Province, 25-VII-2015, collected by Chang-Fa Zhou; 1 male imago, 10 female imagos, Changli Village, Pangquangou Town, Jiaocheng County, Luliang City, Shanxi Province (111.29085° E, 37.51583° N), 24-VII-2006, collected by Dong Liu, Zhao-Feng Wang; 12 nymphs, Hun He, Liaoning Province, 1-IX-2014, collected by Yuan Zhang, Xin Gao; 10 nymphs, Taizi River, Liaoning Province (123.732° E, 41.031° N); 30 nymphs, Lijiagou, Luya Mountain, Ningwu County, Shanxi Province (111.55413° E, 38.41589° N), 20-VII-2006, collected by Dong Liu, Zhao-Feng Wang.

### 3.5. Serratella setigera (Bajkova, 1967)

*Ephemerella setigera* Bajkova, 1967: 333 [54], figures 7–8 (nymph). Holotype: Nymph, from Russia.

*Ephemerella (Serratella) setigera*: Gose, 1980: 366 [55], figure 23 (nymph); Yoon and Kim, 1981: 39 [56], figures 7, 55, 61 (nymph); Gose, 1985: 28 [57], figure 120 (nymph); Tshernova et al., 1986: 138 [30], figure 61 (male). 

*Ephemerella setigera*: Okazaki, 1984: 21 [58], figure 31 (egg); Kluge, 1995: 42 [48]; Ishiwata, 2000: 75 [59], figures 23–25 (nymph, male); Ishiwata, 2001: 63 [27].

*Serratella setigera*: Tiunova and Belov, 1984: 74 [60], figure 1 (male, female); Yoon and Bae, 1988: 31 [61], figures 27–28 (nymph); Bauernfeind and Soldán, 2012: 474 [7]; Zhou, 2013: 182 [29]; Zhou, 2015: 230 [25].

*Ephemerella (Torleya) setigera*: Kluge, 1997: 213 [10], table 18. figures 7–8 (nymph); Kluge, 2004: 313 [11].

*Serratella setigera*: Quan, et al., 2002: 249 [23], figure 19 (nymph) (first record from China); Jacobus and McCafferty, 2008: 242 [6]; Tiunova and Bazova, 2010: 328 [62]; Potikha, 2015: 26 [63].

Distribution: China (Northeast), Russia, Korea, Japan.

Descriptions: See Bajkova, 1967 [54], and Tshernova et al., 1986 [30].

Diagnosis: The nymphs of this species have short, paired abdominal tergal spines with stout, blunt bristles (Figure 1K,L). The nymphal bodies are relatively flat (Figure 1J), the maxillary palpi are short (Figure 8I–K), and the apical segments of the labial palpi (Figure 8L) are longer than those of *S. brevicauda* (Figure 8D) but similar to those of *S. acutiformis* **sp. nov.** (Figure 2H). The male imagos of this species have membranous portions between the penes lobes and have prominent dorsal apical projections (Figure 6G–I).

Remarks: The caudal filaments of this species’ nymphs have spine-like bristles only on the articulations (Figure 9E). This characteristic is the same as *S. zapekinae* (Figure 9F), but the other species in this study have additional lateral hair-like setae (Figure 4E and Figure 9C,D).

Materials examined: 17 male imagos, with 4 nymphs, Chuoer River, Talqi Town, Yakeshi City, Inner Mongolia Province (121.11288° E, 47.58584° N), 4-VIII-2007, collected by Chang-Fa Zhou, Hui Xie, Shi-Lei Wang; 1 male imago and 1 male subimago, Chuoer River, Talqi Town, Yakeshi City, Inner Mongolia Province (121.11288° E, 47.58584° N), 3-VIII-2007, collected by Chang-Fa Zhou, Hui Xie, Shi-Lei Wang; 1 nymph, Niba Mountain, Yingjing City, Sichuan Province (102.8674131° E, 29.7254538° N), 17-VI-1996, collected by Chang-Fa Zhou; 2 female imagos and 2 male subimagos, Yulin Forest Farm, Weihe Town, Shangzhi City, Heilongjiang Province (128.0931976° E, 44.0028609° N), 16-VII-1993, collected by Li-You Wen, Sun-Chang Hai.

### 3.6. Serratella zapekinae Bajkova, 1967

*Ephemerella naz* Imanishi, 1940: 206 [64], figure 16 (nymph, from China) (named as *Ephemerella zapekinae* by Bajkova, 1967: 329 [54], figures 3–5, nymph and imago).

*Serratella zapekinae*: Tiunova, 1984: 46 [60]; Zhou et al., 1997: 125 [19], figure 5; Bae and Yoon, 1997: 46 [65]; Quan et al., 2002: 249 [23], figures 20, 70, 88, 123, 186, 201 (nymph, probably a misidentification); Jacobus and McCafferty, 2008 [6]: 242; Bauernfeind and Soldán, 2012: 474 [7]; Potikha, 2015: 26 [63].

*Ephemerella (Serratella) zapekinae*: Tshernova et al., 1986: 138 [30], figure 62 (male); Bae and Soldán, 1997: 147 [7]; Bae and Andrikovics, 1997: 157 [66].

*Ephemerella zapekinae*: Kluge, 1995: 44 [48] (holotype and paratypes); Kluge, 2004: 313 [11]; Soldán et al., 2009: 663 [67].

*Ephemerella (Torleya) zapekinae*: Kluge, 1997: 213 [10], figure 18: 13 (nymph); Kluge, 2009: 132 [68].

Distribution: Most of China, Mongolia, Russia, Korea.

Diagnosis: See diagnoses for *S. ignita* and *S. acutiformis* **sp. nov.** and remarks under *S. setigera*. This species can be recognized in the nymphal stage by the protuberances on the head (Figure 9A) and pronotum. Its caudal filaments have no lateral hair-like setae (Figure 9F). Its penes have no prominent projections (Figure 10H–J).

Materials examined: 15 male imagos, Huma River Bridge, Tahe County, Heilongjiang Province (124.41934° E, 52.18273° N), 16-VIII-2007, collected by Shi-Lei Wang, Hui Xie; 5 nymphs, Mount Li National Nature Reserve, Yuanqu County, Shanxi Province (111.58314° E, 35.23305° N), 1-VIII-2006, collected by Dong Liu, Zhao-Feng Wang; 1 nymph, Great Mountain National Forest Park Shangsi County, Fangchenggang City, Guangxi Province (107.54169° E, 21.54232° N), 18-VII-2005, collected by Peng Li, Dong Liu; 8 nymphs, Yalu River, Boketu Town, Yakeshi City, Inner Mongolia Autonomous Region (121.54724° E, 48.44625° N), 2-VIII-2007, collected by Chang-Fa Zhou, Hui Xie, Shi-Lei Wang; 8 male imagos, Chuoer River, Talqi Town, Yakeshi City, Inner Mongolia Province (121.11288° E, 47.58584° N), Shi-Lei Wang, 4-VIII-2007, collected by Chang-Fa Zhou, Hui Xie.

## 4. Discussion

This detailed research allows us to make some generalizations about the genus *Serratella* in China. All known Chinese *Serratella* nymphs share the following combination of characters that distinguish them from other ephemerellid genera: ventral lamella of gill VI deeply cleft; gills III not operculate, nor distinctly semi-operculate; tarsal claw with only a single row of denticles, in which the most distal denticle is not very much larger than the rest; and abdominal terga with paired posteromedial projections that are situated subparallel to one another. A tentative diagnosis can be made for those Chinese *Serratella* species with male imagos that have been described: genital forceps segment II is somewhat compressed, with a slight apical twist; and the penes have dorsolateral projections and/or obvious spine-like setae variously situated laterally and/or in the apical cleft. Eggs are not consistently distinguishable from all other Ephemerellidae genera, but they usually have a smooth chorion and often have a nipple-shaped polar cap.

Edmunds [1] separated the genus *Serratella* from *Ephemerella* using imaginal dorsal penial projections and the nymphal setae pattern of the caudal filaments, the former genus having projections on penes but lacking lateral setae on nymphal tails. However, the Chinese species, including *S. acutiformis* **sp. nov.** and *S. brevicauda*, show a mix of these characters, having setae on both the articulations and lateral sides of the caudal filaments (Figure 4E and Figure 9C), and the penes of *S. acutiformis* **sp. nov.** have dorsal projections (Figure 6C). On the other hand, the Chinese species in the present study also show that, when present, the size of dorsal penial projections is variable, with those of *S. fusongensis* being small (Figure 6E) and those of *S. acutiformis* **sp. nov.** being larger (Figure 6B); two of the species lack prominent projections altogether. So, in order to clarify the exact relations between Palearctic and Nearctic *Serratella* species (the latter group containing the type species of the genus), more direct and detailed comparisons of species within the genus and with species in other genera are needed, which may result in modifications to the definition of the genus *Serratella*.

Jacob [5] suggested that *Serratella* nymphs have reduced maxillary palpi and no lateral hair-like setae on caudal filaments and that imaginal penes have dorsal projections. In contrast, *Ephemerella* was said to have hair-like setae on tails, maxillary palpi relatively well developed, and male imagos with penes lacking dorsal projections. Based on these characters, he put the species *E. ignita* in the genus *Serratella*. Using the same characters, on the contrary, Bauernfeind and Soldán [7] retained this species in the former genus. Furthermore, Edmunds [1] and Allen and Edmunds [32] realized that some species of *Ephemerella* and *Serratella* have spines on the lateral margins of the penes (such as *Serratella serrata* Morgan, 1911 [2]), but Bauernfeind and Soldán [7] defined the genus *Serratella* without spine on penes. For the same reasons mentioned above (these characters are variable and Chinese species show intermediate states between them), we think we should consider other candidate characters to separate those two genera.

Kluge [10,11] and Jacobus and McCafferty [6] adopted the character of gills VI to delimitate the genera *Serratella* and *Ephemerella*. *Serratella* has a deeply cleft ventral lamellae of gill VI, while *Ephemerella* lacks this deep cleft. Given this, the species *E. ignita* was included in the genus *Serratella*. This kind of classification allows not only a compromise with respect to some of the apparently paradoxical characters described above but it also retains both *S. ignita* and *S. zapekinae*, whose penes are extremely similar to each other but different from those of other *Serratella* species, in the genus *Serratella*. Therefore, it is followed in the present study.

Unfortunately, this classification causes another problem. Both the genera *Serratella* and *Torleya* have similar gills VI, and their males usually have dorsal penial projections and similar forceps. Further, the gills III of many *Torleya* species are not enlarged, such as those of *Torleya mikhaili* Tiunova, 1995 [34], which is a key character used to separate them, originally by Edmunds [1]. Kluge [10,11] regarded those two genera as one, and Edmunds [1] did express reservations about their distinctiveness. A comprehensive and comparative study of these species and a few in some other genera is needed to resolve problems regarding identification and classification globally. At this moment, however, when we consider just the Chinese materials, we see that the nymphs of *Torleya* have more hair-like setae but fewer and smaller abdominal tergal spines than those of *Serratella*, and the male imagos have slight differences in the apical segments of their forceps.

## Figures and Tables

**Table 1:** Fifteen Names of *Serratella* Species in China.

Chinese Species Recently Included in *Serratella*	Current Status	References
*Serratella ignita* (Poda, 1761)	*Serratella ignita* (Poda, 1761)	[6,16]
*Serratella zapekinae* Bajkova, 1967	*Serratella zapekinae* Bajkova, 1967	[19]
*Serratella setigera* Bajkova, 1967	*Serratella setigera* Bajkova, 1967	[23]
*Serratella longipennis* Zhou et al., 1997	*Serratella longipennis* Zhou et al., 1997	[18]
*Serratella brevicauda* Jacobus et al., 2009	*Serratella brevicauda* Jacobus et al., 2009	[24]
*Serratella fusongensis* Su and You, 1988	*Serratella fusongensis* Su and You, 1988	[15,25]
*Serratella hainanensis* She et al., 1995	*Teloganopsis jinghongensis* Xu et al., 1984	[6,17,26]
*Serratella tumiforceps* Zhou and Su, 1997	*Torleya nepalica* (Allen et Edmunds, 1963)	[20,28]
*Serratella longforceps* Gui, Zhou and Su, 1999	*Torleya longforceps* (Gui et al., 1999)	[6,21]
*Serratella albostriata* Tong & Dudgeon, 2000	*Teloganopsis jinghongensis* Xu et al., 1984	[6,22,26]
*Ephemerella (Serratella) jinghongensis* Xu et al., 1984	*Teloganopsis jinghongensis* Xu et al., 1984	[6,13]
*Ephemerella (Serratella) rufa* Imanishi, 1937	*Teloganopsis punctisetae* (Matsumura, 1931)	[6,27]
*Ephemerella (Serratella) nigromaculata* Xu et al., 1980	*Cincticostella gosei* Allen, 1975	[12,25,29]
*Ephemerella (Serratella) tianmushanensis* Xu et al., 1980	*Cincticostella gosei* Allen, 1975	[25,29]
*Ephemerella (Serratella) xiasimaensis* (You, 1987)	*Serratella xiasimaensis* (You, 1987)	[14]

## Data Availability

All data are available in the paper.
